# Novel Technique for Box-Lesion Ablation of Atrial Fibrillation Combined with Off-Pump Coronary Surgery

**DOI:** 10.21470/1678-9741-2022-0146

**Published:** 2023

**Authors:** Aleksandr Zotov, Daniil Borisov, Aleksandr Troitskiy, Robert Khabazov

**Affiliations:** 1 Department of Cardiac Surgery, Federal Research Clinical Centre, Moscow, Russia; 2 Adult Congenital Heart Disease Service, Liverpool Heart and Chest Hospital, Liverpool, United Kingdom

**Keywords:** Atrial Fibrillation, Constriction, Coronary Artery Bypass, Off-Pump, Coronary Artery Disease, Coronary Vessels, Electrodes, Equipment and Supplies

## Abstract

**Introduction:**

We propose a new technique for box-lesion ablation combined with off-pump coronary artery bypass grafting for the treatment of patients with coronary artery disease and paroxysmal or persistent atrial fibrillation.

**Methods:**

Eight male patients with paroxysmal (n=2) or persistent atrial fibrillation (n=6) and coronary artery disease underwent box-lesion ablation combined with off-pump coronary artery bypass grafting. Box-lesion ablation was performed using a bipolar flexible clamping device with irrigated electrodes which was originally designed for thoracoscopic epicardial ablation.

**Results:**

Complete revascularization was performed in all patients. There were no deaths or major complications. At a median follow-up of 14 months, seven patients (87.5%) were in sinus rhythm.

**Conclusion:**

Box-lesion ablation can be easily and effectively combined with coronary artery surgery in an off-pump setting.

## INTRODUCTION

Atrial fibrillation (AF) is common in many patients presenting for cardiac surgery^[1]^. Data from the Society of Thoracic Surgeons indicates that AF is present in 5.1% of patients requiring coronary artery bypass grafting (CABG)^[2]^. Nevertheless, concomitant ablation was performed in only 33.0% of patients undergoing isolated CABG^[2]^. The development of technically more convenient techniques used in beating heart surgery may support a wider introduction of AF ablation in CABG patients.

In some patients, beating heart exposure of pulmonary veins (PVs) results in haemodynamic instability and that is why we offer to use a flexible ablation device (Cardioblate™ Gemini™-S). This device is widely used in thoracoscopic procedures with promising mid- and long-term results^[3-5]^.

## METHODS

### Study Population

From August till December 2019, eight male patients underwent surgical off-pump revascularization with concomitant box-lesion ablation and left atrial (LA) appendage resection. All patients had symptomatic coronary artery disease (CAD) and paroxysmal (PaAF) or persistent atrial fibrillation (PeAF). No other cardiac surgical procedure was performed.

Patients’ baseline characteristics are summarized in Table 1.

**Table 1:** Baseline characteristics of the patient population (n=8).

Male, n (%)	8 (100%)
Paroxysmal atrial fibrillation, n (%)	2 (25%)
Persistent atrial fibrillation, n (%)	6 (75%)
Age, years, median (min-max)	67 (59; 75)
CHA₂DS₂VASc, median (min-max)	2 (1; 4)
Duration of atrial fibrillation, months, median (min-max)	26 (5; 42)
Previous history of acute myocardial infarction, n (%)	6 (75%)
Previous history of thromboembolic events, n (%)	2 (25%)
Stroke	1
Transient ischemic attack	1
Body mass index, kg/m^2^, median (min-max)	30 (24; 35)
Left atrial diameter, mm, median (min-max)	43 (35; 49)
Indexed left atrial volume, ml/m^2^, median (min-max)	55 (42; 67)
Left ventricular ejection fraction, Simpson, %, median (min-max)	50 (42; 63)
Pulmonary artery pressure, mmHg, median (min-max)	36 (24; 52)
Hypertension, n (%)	6 (75%)
Chronic obstructive pulmonary disease, n (%)	2 (25%)
Diabetes, n (%)	3 (37.5%)
Glomerular filtration rate < 60 mL/min/1.73 m^2^, n (%)	2 (25%)

CHA₂DS₂VASc=congestive heart failure, hypertension, age ≥ 75 (doubled), diabetes, stroke (doubled), vascular disease, age 65 to 74, and sex category (female)

The study protocol was approved by the Local Ethics Committee (P19/0021), and all patients signed informed consent forms prior to surgery.

### Surgical Procedure

After median sternotomy, the conduits were harvested, and heparin was administered. Left internal mammary artery and saphenous vein were used as grafts.

**Fig. 1:**
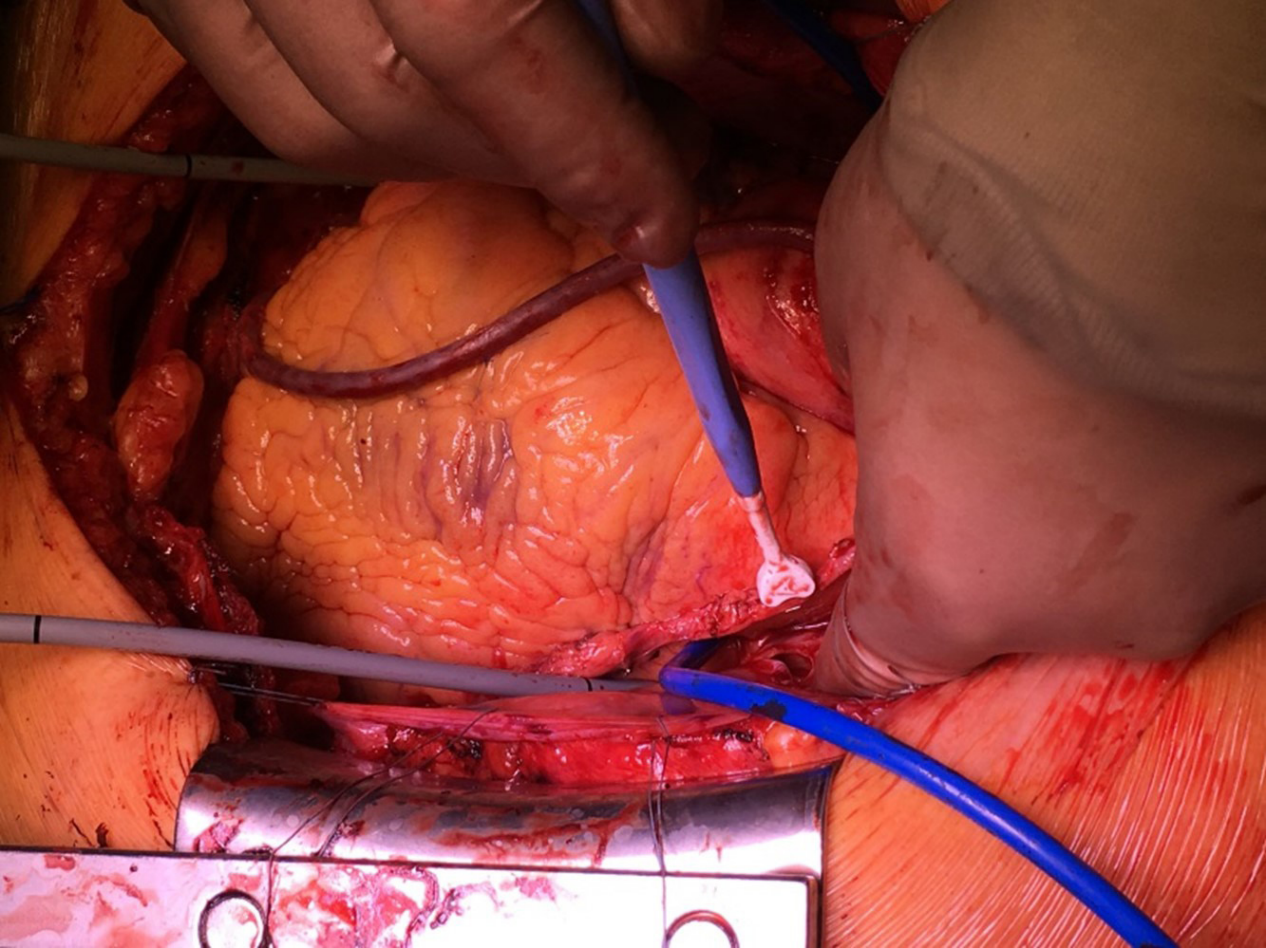
Placement of catheters. Left internal mammary artery flow measurement.

Coronary revascularization was performed before ablation using tissue stabilizers and apical suction positioning devices. Proximal anastomoses were performed using Heartstring® Proximal Seal System. On completion of grafts, intraoperative graft flows were assessed using a flowmeter (Figure 1).

The ablation was performed using a flexible bipolar irrigated ablation device - Cardioblate™ Gemini™-S. The device was designed for thoracoscopic procedures as was described by Doty et al.^[3]^ in the original paper.

Blunt dissection was performed to open the transverse sinus by entering the space between the right pulmonary artery and the right superior PV (posterior to the junction of the superior vena cava and right atrium). The oblique sinus (the space between the right inferior PV and the inferior vena cava) was opened in the same fashion.

Two rubber catheters were passed through the sinuses behind the left atrium, above and below the superior and inferior PVs to guide the device clamps (Figure 1). The clamps were then attached to ends of the catheters outside the wound.

**Fig. 2:**
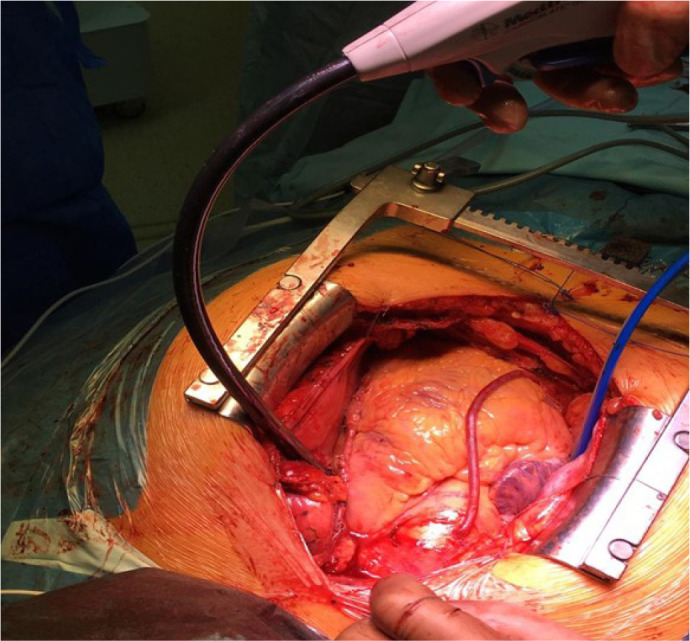
Formation of left half of pulmonary vein encircling lesion.

**Fig. 3:**
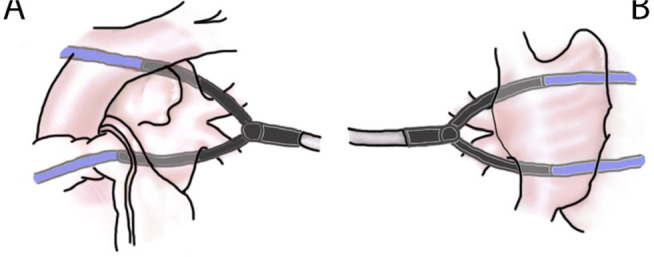
Scheme demonstrating the position of electrodes during the ablation. Guiding catheters (blue colour) are attached to electrodes.

Gentle traction on ends of the catheters was used to draw the device into position across left PVs and left atrium. Once appropriately positioned, it was clamped over the left half of the left atrium (Figures 2, 3A). Ten lesions were applied guided by impedance drop to confirm transmurality. After each application, the device was adjusted a little to get overlapping lesions. The process was then repeated on the right side - hereby creating a box lesion (Figure 3B).

**Fig. 4:**
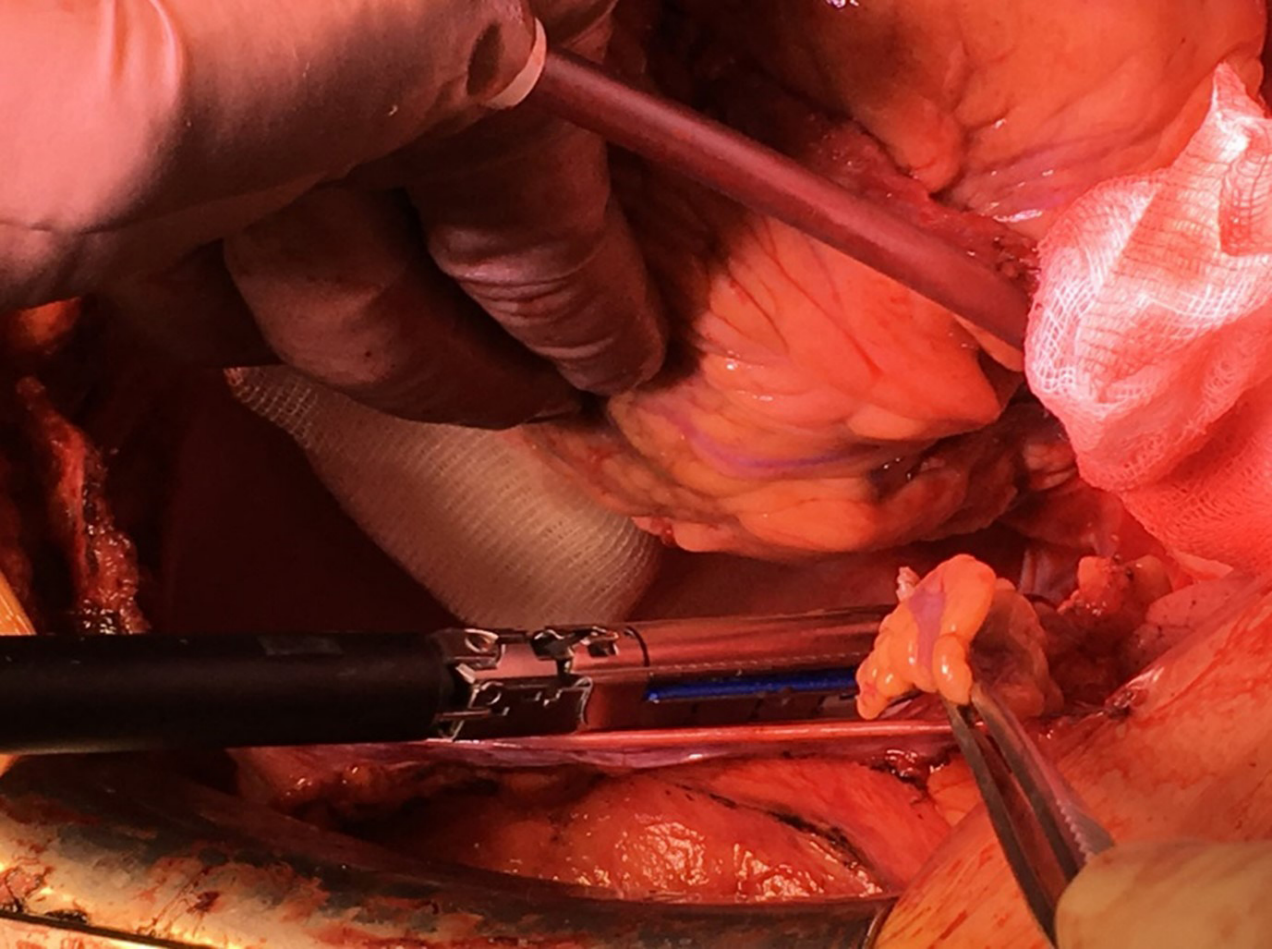
Left atrial appendage resection with a stapler.

The LA appendage was resected with an Echelon™ or Endo GIA™ stapler (Figure 4).

Afterwards, electrical cardioversion was used to restore sinus rhythm and bilateral epicardial stimulation was performed to check the block of conduction. Exit block was confirmed by pacing the PVs and nonablated tissue within the box after cardioversion with a maximum output of 25 mA. In case of ventricular capture, additional ablation was performed until exit block could be confirmed.

### Postoperative Management and Follow-up

Statins, antihypertensive drugs (if required), and bisoprolol were taken routinely after surgery. Amiodarone was administered intravenously before extubation, and this was followed by amiodarone taken orally for a period of six months. If a patient was in sinus rhythm and symptoms free, antiarrhythmic drugs were stopped after six months. Low-molecular-weight heparin was administered at a prophylactic dose, starting the day after the procedure until the day of discharge. Afterwards, low-molecular-weight heparin was replaced with dabigatran.

All patients were monitored continuously for arrhythmias in intensive care unit. Twenty-four-hour Holter monitoring and transthoracic echocardiography were performed at three, six, and 12 months postoperatively. Recurrence was defined as any electrocardiographically documented episode of AF, atrial flutter, or atrial tachycardia lasting > 30 seconds occurring beyond a three-month blanking period. Patients were encouraged to contact a doctor in case of symptoms of AF or previously unrecognized symptoms. Stress echocardiography was performed at 12 months to confirm freedom from CAD.

Generally, dabigatran was discontinued after six months in the absence of atrial tachyarrhythmia recurrences unless prior thromboembolism or other indication for chronic anticoagulation.

### Statistical Analysis

Microsoft® Excel 2010 was used for statistical data analysis. The data were presented as median and range or number and percentage.

## RESULTS

There was no operative mortality, no myocardial infarction, and no stroke nor transient ischemic attack. Two patients with PaAF were in sinus rhythm before the procedure. Electrical cardioversion on the operating table was successful in four patients with PeAF. Conduction block was confirmed in all patients with sinus rhythm.

Detailed periprocedural data and complications are shown in Table 2.

**Table 2:** Periprocedural characteristics (n=8).

Surgery time, minutes, median (min; max)	190 (142; 247)
Ablation time, minutes, median (min; max)	18 (16; 23)
Number of grafts per patient, median (min; max)	3 (2; 4)
Left atrial appendage resection, n (%)	8 (100%)
Intubation time, hours, median (min; max)	5 (4; 8)
Time with chest tubes, hours, median (min; max)	24 (14; 70)
Postoperative blood loss, ml, median (min; max)	350 (250; 750)
Intensive care unit stay, hours, median (min; max)	18 (14; 32)
Hospital stay, days, median (min; max)	8 (5; 14)
Complications, n (%)	2
Need for blood product transfusion	1
Pleural effusion requiring drainage	1

In two patients with PeAF, sinus rhythm was restored within 24 hours, after amiodarone infusion and electrical cardioversion. AF recurred on postoperative day four in one patient, and the sinus rhythm was restored after electrical cardioversion.

All patients underwent a control examination three, six, and 12 months after the procedure.

At a median follow-up of 14 months (range 12-17), there were no signs of recurrence of AF nor other supraventricular arrhythmia in seven patients (87.5%). AF recurred in one patient who had long-standing PeAF before the surgery. The form of AF in this patient shifted to paroxysmal. Therefore, amiodarone was used for rhythm control.

Stress echocardiography at 12 months showed freedom from CAD in all patients.

## DISCUSSION

This is the first study showing safety and feasibility of box-lesion ablation with the use of a flexible clamping device during off-pump CABG (OPCABG).

Previous studies have shown that surgical ablation of AF concomitant with bypass surgery provides improved long-term survival^[6]^. In randomized controlled trials and matched patient populations, surgical ablation of AF during cardiac surgery proved to be safe, effective at restoring sinus rhythm, and associated with late improved exercise capacity and survival compared to untreated patients^[2,6]^.

The recent guidelines gave Class I recommendations for the concomitant surgical ablation of AF without additional risk of operative mortality or major morbidity^[7,8]^. They recommended surgical AF ablation at the time of concomitant CABG (Level B) to restore sinus rhythm^[7,8]^. Although the Cox-Maze procedure remains the gold standard for surgical treatment of AF, the necessity of performing a Maze procedure for all AF patients is a debatable issue. The downside of such procedure is opening of the left atrium for ablation which requires cardiopulmonary bypass. In those patients who do undergo surgical AF ablation, the most commonly performed ablation procedure is PV isolation, simply because of technical difficulties when faced to do more^[9]^. Technical issues can be encountered in patients with a low left ventricular ejection fraction or an enlarged heart, as these patients may not tolerate the procedure hemodynamically. OPCABG is already a challenging procedure in such patients, and further manipulating the heart during ablation and pacing may be problematic.

Intraoperative hypoperfusion may result in organ ischemic damage and increase the risk of postoperative complications such as stroke or acute renal insufficiency^[10]^. The use of flexible ablation device helps to overcome these difficulties.

We believe that the described technique can be successfully combined with conventional on-pump CABG and minimally invasive CABG (in this scenario some steps of ablation procedure are performed thoracoscopically). Importantly, the use of this technique does not require intensive training and high level of surgical skills.

At the same time, we need to admit that box-lesion ablation cannot be effectively used in patients with pulmonary artery hypertension (pulmonary artery pressure > 45 mmHg), prolonged history of AF (> 5 years), prominent LA dilatation (indexed LA volume > 48 ml/m^2^), or significant mitral valve disease. In these groups of patients, concomitant Cox-Maze IV procedure would be preferrable.

### Limitations

The main limitation of the study is a small sample size. Nevertheless, the main purpose of our work was to show feasibility of the surgical technique described. Further studies aiming to describe effectiveness will follow.

## CONCLUSION

Box-lesion ablation can be safely and effectively combined with coronary artery surgery in an off-pump setting. It may be useful in selecting the best ablation approaches for high-risk patients with AF and CAD.
